# Development and Exploration of Psychometric Properties of the Family Adjustment Questionnaire for Admitting an Older Adult to a Nursing Home (CAFIAR)

**DOI:** 10.3390/ijerph17207597

**Published:** 2020-10-19

**Authors:** Antonio Riquelme-Marín, Marta Martín-Carbonell, Juan M. Ortigosa-Quiles, Inmaculada Méndez

**Affiliations:** 1 Department of Personality, Assessment and Psychological Treatments, University of Murcia, 30100 Murcia, Spain; ortigosa@um.es; 2 Faculty of Psychology, Universidad Cooperativa de Colombia, Santa Marta 470006, Colombia; martha.martinc@campusucc.edu.co; 3 Department of Evolutionary and Educational Psychology, University of Murcia, 30100 Murcia, Spain; inmamendez@um.es

**Keywords:** family adjustment, nursing home, questionnaire development, psychometric properties

## Abstract

*Background*: Admitting an older adult to a nursing home involves significant adjustment efforts by the family. Our goal was to prepare an assessment instrument for this, given that there was none to date. *Method*: Participants—134 relatives from different nursing homes in the region of Murcia. Instruments—structured interview for socio-demographic information, satisfaction with the nursing home, well-being and health self-assessment, Radloff’s Depression Questionnaire (CES-D), and the first version of CAFIAR. *Results*: A 15-item instrument with three factors was obtained: Factor 1 (Unease due to admitting an older adult to a nursing home), Factor 2 (Relief), and Factor 3 (Nostalgia and concern for the older adult), in addition to a general adjustment index, with a Cronbach’s alpha of 0.74. The general adjustment index and the subscales that demonstrate poor adjustment were significantly correlated with depression and a worse health self-assessment, while the Relief subscale, which indicates better adjustment, was significantly correlated with well-being and a positive health self-assessment. *Conclusions*: The family adjustment in admitting an older adult to a nursing home questionnaire (CAFIAR) has adequate psychometric properties to assess family adaptation in admitting an older adult to an institution.

## 1. Introduction

The increase in individual and population aging since the 20th century has resulted in great challenges for nations, including the fact that there will be more people needing support and care in old age that neither they nor their families will be able to satisfy.

According to WHO data, between 2015 and 2050, the percentage of the world population over 60 years will increase from 900 million to 2 billion, which represents an increase from 12% to 22% [1]. This prediction is particularly concerning, taking into account that we are not only assisting an aging population in a generalized manner but also that the senior population sector presents an increased advanced age and level of care needed due to functional dependence.

Most studies related to caring for seniors with serious dependence issues indicate that families prefer informal care for the dependent older adults at the community level [2,3]. However, some studies indicate that in Western societies, between 75% and 90% of the people with serious problems that are worsening are admitted to a nursing home as a result of the family’s inability to take over their care [4,5].

Nursing homes have been one of the oldest resources used as a solution to caring for seniors with partial or total loss of autonomy. Despite all the inconveniences nursing home admission may cause, it is necessary to take into account that it is the most used or requested assistance support resource for older adults with serious dependence issues.

Admission to a nursing home implies an abrupt change in the older adult’s lifestyle, undoubtedly requiring major adjustment efforts. According to Kydd [6], the loss of a familiar environment and people they know as well as significant lifestyle modifications may be associated with the anger, depression, or defensiveness of the newly admitted resident, who may refuse to interact with others. There are many studies related to the psychological impact of admitting an older adult to a nursing home [7,8,9] as well as the factors related to the person’s adjustment [10,11]; however, there are few studies on the impact of institutionalization on the relatives of the older adult who has been admitted.

Occasionally, admission to a nursing home is preceded by specific events. These events may be directly related to the caregiver, as in the case of hospitalization or serious illness [2,12] or to the older adult, such as sudden changes in self-care abilities or sudden loss of autonomy, whether due to physical or psychological difficulties [4,8].

In other cases, factors related to admission are not caused by unexpected events but are related to the worsening of behavioural problems, such as aggression, incontinence, mobility, and increasing deterioration in people with dementia [13].

As stated by Sury et al. [4], the decision to admit a spouse or another relative to a nursing home has important emotional effects on caregivers. These emotional responses may be related to guilt, sense of failure, sadness, relief, or loss. In relation to this loss, some studies [9,14] have indicated that, particularly among wives, a period of mourning may arise upon admission, sometimes preceded by pre-mourning in cases of prior cognitive deterioration.

Admitting an older adult to a nursing home as a form of family abandonment generally constitutes a false myth that may exert added pressure on those who are closest to the adult [15,16]. Against this prototype, research on the psychological impact on families after a nursing home admission indicates that sadness and guilt are frequent manifestations related to institutionalization. In fact, admission to a nursing home, far from implying abandonment by the relative, generally entails the gradual assumption of a new role in providing care [17].

In different studies, it has been shown that, after an older adult is admitted to a long-term care institution, the family continues to experience feelings of burden [18,19], while feelings of guilt [20] or depression [14] may arise.

The importance of certain factors as families adapt to institutionalization, such as the senior’s involvement in making said decision, professional support during decision-making, agreement from other family members and society, as well as aspects related to satisfaction with the nursing home’s function and interaction and collaboration with the formal care system [21,22,23], has been pointed out. However, little effort has been made to include these factors in a theoretical model to help us better understand the family adjustment process.

A pioneering initiative that explains family adjustment to admitting older adults to nursing homes was proposed by Rosenthal and Dawson [24,25]. Initially, their model is the result of a pilot study, including in-depth interviews with 14 wives of institutionalized men. Interviews were retrospective and focused on the wives’ feelings, actions, and perceptions concerning institutionalization, the factors that contributed to or made it difficult to find solutions to the problems encountered and how and when they were solved. This adjustment process was called “pseudo-widowhood”, in reference to “losing” their spouses, although they were still alive.

The authors’ theoretical development is based on a sequential perspective divided into four stages: The first one, known as ambivalence/insecurity, is characterized as an exhausting phase at the physical and emotional levels and is filled with concerns, feelings of depression, demoralization, loneliness, solitude, resentment, and deterioration of the sufferer’s own health. The second stage is defined by frequent visits to the nursing home. It entails difficulties in adapting to their new role of caregiver, as their visits to the centre increase several times over to monitor the tasks performed, and they may over-care and hyper-devote themselves to take compensatory care for the older adult. The third phase is marked by reallocation, as they relinquish some roles and adapt to cooperate with the formal care system. Finally, the fourth stage involves resolution and adaptation, achieving greater balance and accepting the new reality and the role that the family can play.

More recently, Butchers’ team [19] proposed a theoretical model that attempts to explain the decision-making process regarding admitting an older adult’s dependency issues and the family’s adaptation to this situation.

Four stages of this process are specifically worth emphasizing: The first is characterized by moving towards an inescapable decision. This pattern would begin by gradually understanding that it is becoming increasingly complex to meet the older adult’s needs at home, which will inevitably lead to a point of no return that will require the adult to enter an institution to receive ongoing care. The second element involves struggling with the decision. During this period, expressions describing the situation as “hard”, “difficult”, “terrible”, or “devastating” become particularly relevant. The older adult’s family member deals with the inevitability of having to come to an unwanted decision imposed by reality, often without the involvement of the older adult and not always supported by their closest social environment.

The third pattern described by the authors is “looking for peace and quiet”. According to Butcher et al. [19], “caregivers and family members need to prove they have made the right choice and perceive that their relatives are better cared for at this new place than in their own homes”. Finally, the last pattern involves staying connected. This period may comprise a grief-related component, as they miss the person who is not home anymore. This nostalgia can disappear as they commit themselves to their ongoing care.

The research conducted on the psychological consequences concerning the institutionalization of an older adult in the family has shown that, after their admission, the family keeps on assuming the difficulties related to the older adult’s health problems, in addition to the problems faced during the above decision-making and changes in circumstances. In this regard, it is worth mentioning the different research lines associated with identifying the psychological implications of institutionalization within the family [22], presence of risk factors for admission and subsequent adaptation [21], or development of programmes aimed at incorporating relatives in the formal care system [24]. Nonetheless, for this knowledge area to further develop, measures aimed at properly assessing family adjustment to the older adult’s institutionalization are needed.

The work presented below is the result of a research line developed by our team, whose purpose was to evaluate the concept of adjustment to this relocation type. To this end, a questionnaire of 43 items about family adaptation to institutionalization was initially drafted and developed based on a scientific literature review on this matter.

The studies conducted by Rosenthal and Dawson [25] and Butcher et al. [19] were particularly useful, as the purpose of their questions was to explore feelings of insecurity, depression, demoralization, or resentment (typical of Stage 1); frequency of visits and satisfaction (referred to in Stage 2); patterns of interaction with the technical staff (specific to Stage 3); and feelings of acceptance mentioned in the last stage. In addition, these items included defining elements for the phases described by Butcher’s model, mainly in terms of the aspects related to the impact of the decision adopted and the quest for peace of mind, in addition to the incorporation of 11 items intended to assess features associated with their opinion of the nursing home [26].

Questions were asked to a sample of 82 family members of older adults living in nursing homes in the region of Murcia, Spain, in a structured interview format for establishing their relationship with the time of institutionalization. The results obtained concur with the differences noted in the family impact regarding institutionalization depending on the time elapsed, although they mainly showed that the use of a theoretical framework based on a sequential pattern hampered the most suitable understanding of the mechanisms that may be interacting [26].

These results are in line with those obtained by other studies conducted by our team [27], in addition to the findings provided by other authors [22,28]. Thus, we believe it would be more appropriate to adopt a dimensional approach for the assessment of family adjustment to institutionalization.

The aim of the present paper is to present the development of a questionnaire whose purpose will be to assess the adjustment of the relatives in relation to the elder’s admission to a nursing home, and which will lay on the basis of a multidimensional model of this process. Moreover, both the psychometric properties and instrumental adequacy have been explored within a sample of Spanish family caregivers on the purpose of contributing to the development of new research on this subject. We believe that ensuring the existence of a solid and easily applicable instrument for the assessment of family adaptability may entail an invaluable support for research on topics such as factors associated to admission impact, the helpfulness of programs designed for incorporating family members to the nursing home environment, the user’s own adjustment, and the quality of the relationship between the elder and the family member after the admission. Results deriving from this proposal should redound in direct benefits for family members, but also for the users and care environment. Furthermore, the design of an instrument for family adjustment assessment will necessarily have to answer the research question that lays at the core of the present paper, which is no other than identifying the dimensions that make up for family adjustment regarding the admission to nursing homes as a research construct.

## 2. Materials and Methods

### 2.1. Participants

The study involved the participation of 134 family members, of which 34.3% were men and 65.7% were women. The average time of stay for older adults in the nursing home at the time of the study was 33.09 months (SD = 43.5, mode = 12, min. = 1 month, and max. = 347 months), with 41.4% of the cases being less than 12 months. The participants’ age range was 26–78 years old (M = 51.95 years; SD = 11.13) from three institutions located in Murcia and were selected as they agreed to participate in the study.

All participants were from Spain, with 96.4% of them being residents of the same province where the nursing home is located. Table 1 shows the data corresponding to the socio-demographic characteristics of the study participants.

### 2.2. Measures

Structured interview: This model was developed to obtain socio-demographic information on the older adult’s interviewed relative (sex, marital status, employment status, and education) and their relationship with the institutionalized senior. The interview also included five questions to find out more about the relative’s satisfaction with the nursing home (with two possible answers: very satisfied/not very satisfied), three self-assessment questions regarding their health status (with two possible answers: I agree/I disagree), and five self-assessment questions concerning their well-being (relief, satisfaction, optimism, energy, and happiness). Answers were rated by psychologists and divided into two categories (high level/low level).

CAFIAR—the first version: The first version of the instrument was developed based on the theoretical analysis of the initial set of items, comprising 22 items that the individual had to answer in accordance with a 5-point Likert scale (No, A little bit, Moderately, A lot, and Completely; see the Procedure section to review the development of the Questionnaire’s first version, Step 2: Item selection and qualitative analysis).

Radloff’s Depression Questionnaire (CES-D): Originally developed to measure depressive symptomatology in general population epidemiological studies [14], although it has also been extensively used in different settings as a case-finding measure for depression and as a stand-alone diagnostic instrument. In Spain, it was validated by Soler et al. [29].

### 2.3. Procedure Used to Develop the Questionnaire’s First Version

#### 2.3.1. Construct Identification

In line with the recommendations made by Muñiz and Fonseca [30], the first step involved the identifying the construct to be evaluated: Family adjustment to an older adult’s admission to a nursing home.

In this regard, a theoretical–conceptual review was conducted with regard to each of the aspects supposed to make up this construct in an iterative process during which researchers discussed the components based on the general construct of adjustment to institutionalization. Defining the components also entailed the integration of the contributions from the literature to this area. This process was supported by question-and-answer sessions with experts (professional caregivers working at nursing homes, relatives of institutionalized older adults, psychologists, etc.) to discuss the definition developed and whether they believed there were other relevant components that needed to be incorporated to said definition or not. A consensus decision was taken regarding the construct’s definition and its components. Table 2 describes the construct and its components as well as the general features that the instrument and items should have, based on our definition.

#### 2.3.2. Item Selection and Qualitative Analysis

The preliminary 54-item set used in the abovementioned prior study (26) was analysed by three judges, of recognized prestige in the area of gerontology, who independently rated them on a scale of 0 to 5 based on their relationship with the relative’s adjustment that it sought to evaluate.

Based on the judges’ evaluation, the items that needed to be excluded, as their content was not in line with the theoretical assumptions with regard to family adjustment to older adult’s admission to the nursing home, were identified. This analysis resulted in the first version of the 22-item instrument, shown in Table 3.

#### 2.3.3. Pilot Study

A pilot study was conducted with 20 relatives of seniors from one of the nursing homes participating in the study, to gather information about the level of understanding of the items, response scale, or any other difficulty with regard to implementation. After applying this piloting, we modified the wording of items 19 and 21, which implied comprehension difficulties.

#### 2.3.4. Data Collection Procedure and Ethical Considerations

Questionnaires were applied by two interviewers who were previously trained by the researchers. Instruments were applied heterogeneously on an individual basis.

The involvement of all participants was confidential, anonymous, and voluntary. This research study is based on the ethical principles and recommendations of the “Declaration of Helsinki” (2000), thus observing the general principle that concern for the well-being of participants has prevailed over scientific interests. In addition, it is in accordance with Royal Decree 1720/2007, of December 21, which approves the regulations for the development of Organic Law 15/1999, of December 13, on the protection of personal data.

#### 2.3.5. Statistical Analysis

Descriptive statistics were calculated to summarize the demographic characteristics, examine the percentage of response to the items, and identify those that should be excluded from the final version of the instrument.

The criteria used to exclude items included presentation of the Z scores outside the range +− 3, asymmetry and kurtosis indices greater than −2 +2, and/or factor saturations below 0.30.

The item–item and item–scale correlations as well as the alpha coefficient were examined. Exploratory factor analysis (EFA) was carried out with the Principal Axes vectorization method. The rotation method was direct oblimin. The factor extraction criterion used was the scree test.

The suitability of factor analysis was checked using the Kaiser–Meyer–Olkin (KMO) test [25] and Bartlett’s test of sphericity [26]. For all analyses, *p* < 0.05 was considered statistically significant.

Pearson’s correlation was used to examine the relationship between the scores achieved on the models identified using the EFA and the answers for geriatric home satisfaction, well-being, self-assessment of their health status, and depression.

A t-test was used to assess the significance of differences in the scores of the new version of the instrument, attributable to socio-demographic variables.

Data analysis was performed using the Statistical Package of Social Sciences (SPSS) software version 25, with the license provided by the Cooperative University of Colombia.

## 3. Results

The item response percentage was over 97%. Three items related to blaming the senior and discomfort during visits showed unacceptable asymmetrical and kurtosis values, so they were removed: “I feel bad every time I visit my relative” (asymmetry = 2.035; kurtosis = 3.75); “My relative makes me feel guilty every time I visit” (asymmetry = 3.32; kurtosis = 11.20); and “I feel upset with my relative because of their lack of support in avoiding institutionalization” (asymmetry = 3.79; kurtosis = 15.02).

A preliminary EFA was conducted using the 22-item version. The KMO index and Bartlett’s test demonstrated the validity of using the EFA. Three factors were removed after taking into account our theoretical expectation and the results of the scree plot, but the factors found were confusing: Factor 1 had nine items and mixed questions related to acceptance of the decision (Component 1) with questions exploring the feeling of loss and concern for the relative (Component 2). Factor 2 integrated the five items associated with Component 2 (Relief), whereas Factor 3 grouped eight questions, with several items showing loads lower than 0.30. Similarly, this factor included the three items regarding satisfaction with the relative’s relationship during visits, which had poor asymmetry and kurtosis values and scores outside an acceptable range.

Based on this result, we decided to develop a new version of the questionnaire, removing the items that met the previously established exclusion criteria, leading to a 15-item questionnaire (Table A1 in Appendix A). The Cronbach’s alpha coefficient for the 15-item scale was found to be 0.74.

A new EFA was conducted on this abbreviated version. The relevance of the new EFA showed a satisfactory KMO sampling adequacy index (0.824) and significance according to Bartlett’s test of sphericity (χ^2^(231) = 1050; *p* < 0.001).

**Figure 1:**
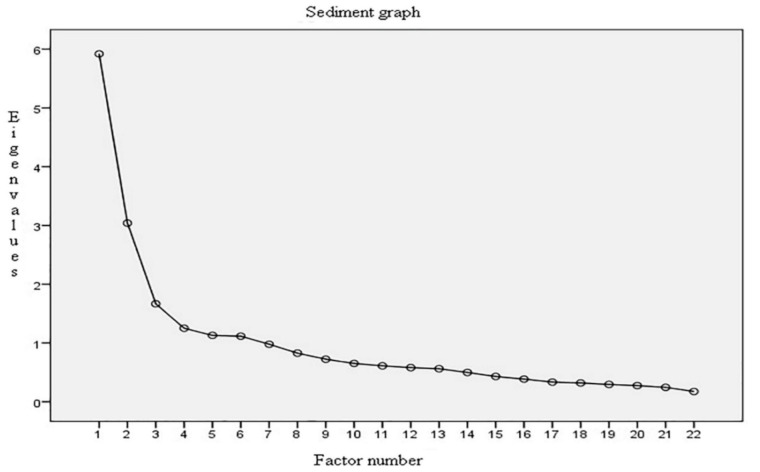
Scree plot of the factor solution.

Once again, the scree plot showed the convenience of extracting the three factors (Figure 1), which agrees with our hypothesis on the construct’s components. These factors represented 51% of the total variance (Factor 1 = 32%, Factor 2 = 15%, and Factor 3 = 4%). Table 4 presents the configuration matrix showing that the three factors obtained are in line with the theoretical expectations.

According to the result of the factor analysis, the three factors (each with five items) were interpreted as subscales and to obtain a simple measure of each subscale, the sum of the ratings were calculated for each subject. The higher scores in Subscales 1 (Unease due to admitting the older adult to the nursing home and 3 (Nostalgia/feeling of loss and concern for the older adult) would suggest poorer adjustment. A general adjustment index (GAI) = (F1 + F3) − F2 was also calculated (the scores of items 21 and 22 must be inverted to calculate the factor 1), which shows a balance between the negative aspects (Dissatisfaction with the decision and the feeling of loss) and the positive aspect (Relief of burden). Therefore, the higher the general index, the poorer is the adaptation.

Table 5 presents correlations between scores in the CAFIAR’s subscales, in its 15-item version, with the score obtained by the CES-D, well-being indicators, health self-assessment, and satisfaction with the nursing home. As expected, the G and the subscales indicating poor adjustment obtained significant correlations with depression and worse health self-assessment, whereas the “Relief” sub-scale, which suggests better adjustment, was found to have significant correlations with well-being and positive health self-assessment. Satisfaction with the nursing home was negatively correlated with scores suggesting poor adaptation, while gratitude towards the staff is significantly and positively correlated with relief.

No significant differences were found between men and women, neither concerning any subscale nor GAI: Factor 1 (t= −0.534; *p* > 0.59); Factor 2 (t = −1.555; *p* > 0.10); Factor 3 (t = 0.429; *p* > 0.06); and GAI (t = 0.926; *p* > 0.30). Neither between the individuals who had a partner and those who did not: Factor 1 (t= 0.255; *p* > 0.80); Factor 2 (t = −1.836, *p* > 0.074), Factor 3 (t = 0.749; *p* > 0.055); and GAI (t = 0.641; *p* > 0.50); or regarding employment (if they worked at home, outside home or were unemployed): Factor 1 (F= 1.59; *p* > 0.20); Factor 2 (F = 0.576; *p* > 0.56); Factor 3 (F = 0.041; *p* > 0.95); and GAI (F = 0.396; *p* > 0.67); or their academic level: Factor 1 (F = 0.328; *p* > 0.72); Factor 2 (F = 0.679; *p*> 0.509); Factor 3 (F = 0.844; *p* > 0.40); and GAI (F = 0.059; *p* > 0.94).

Table 6 shows the descriptive statistics of the subscales and the general index. As can be observed, the acceptance indicators (low score) prevail for Subscales 1 and 3.

## 4. Discussion

This work created an instrument that can assess family adaptation to admitting a senior to an institution and providing specific details about what this adjustment entails, theoretically and operationally. Despite the existence of various instruments to assess family caregivers [31], these target those who live with older adults with severe functional limitations in their own homes. To date, there is no instrument geared towards the family members of older adults who, for several reasons, had to move to long-term care facilities.

The recommendations made by Muñiz (2019) [30] and different international associations [32,33] with regard to the aspects to be taken into account to propose a new instrument have been followed. Therefore, the first step involved the conceptual and operational delimitation of the construct “family adaptation to admitting an elder to an institution”, for which an analysis and comprehensive review of the published literature in this regard were conducted. We are also supported by expert consultation, understanding experts to be the relatives of older adults, professional workers at nursing homes, and psychogerantology researchers. Furthermore, the requirements for the instrument’s application were identified, in addition to the items’ type, number, length, content and distribution, specifications, and instructions on modes and contexts of application. Said information was summarized in a specification chart regarding the construct and items that attempt to represent it, as recommended by the International Test Commission [33]. As has been acknowledged by various authors [34,35], publications on the development of measurement instruments addressing these aspects are scarce, although they are deemed crucial by international standards [34]. Thus, we believe our study will be a great contribution in this regard.

The EFA has been chosen as the statistical procedure implemented to define the instrument’s structure, as it allows for the exploration of the dimensionality of measures and development of internal validity evidence, especially in the initial stages of instrument development or adjustment [35,36]. The principal axis model has been used for factor removal since, according to Lloret [37], “*... it works well when working with small samples, even when the number of variables is high*” (p. 1160).

This procedure, along with the descriptive statistical analysis, helped reduce the number of items and propose a short 15-question version, with proper internal consistency, which comprises three factors.

The first factor obtained is composed of elements referring to the feeling of guilt/acceptance of the decision made. This dimension is closely related to the pattern of “struggling with the decision”, indicated by Butcher et al. [19]. Similarly, it highlights the egodystonic emotions elicited after admission, such as sadness, feelings of failure, guilt, and shame [28].

The first factor, associated with guilt/acceptance, becomes especially relevant not only in our results but also in the discomfort experienced by the family, which sometimes may be due to lack of social support, the myth that institutionalizing is abandoning, lack of specialized support in decision-making, or doubts regarding the benefits the new place may bring for the senior’s care [11,14,20].

The second factor obtained is formed by 5 of the 15 items in the final questionnaire model and refers to the concept of relief, with the items involved being highly homogeneous in content: “I perform better in my day-to-day tasks”, “I have enjoyed returning to the activities I was not able to do before”, “I feel like I can make more time for myself”, “I have enjoyed doing new activities”, and “I feel relieved”. This second factor, positive in terms of institutionalization adjustment, brings to light that this theoretical construct is not only constituted by dimensions of varying substantive nature, but that these parts may have varying valence with respect to adaptation. In addition, this dimension may help understand that different studies may not reach general agreement on the consequences of institutionalization for the family, given that this decision may jointly imply positive consequences, related to relieving the burden, and negative effects, associated with the negative impact that making this decision may also generate.

Finally, the third factor arising from the exploratory analysis also has negative valence and refers to the concept of loss, along with concern for the older adult’s well-being. In this regard, items such as “I miss my relative” or “I wish my relative could come back home” are clear examples of experiences associated with grief over the loss of the preceding situation, whereas “I believe my relative’s health will worsen in the short term” and “I think that my relative’s health will worsen due to staying in a nursing home” are models of the concern about the senior.

The element of loss and nostalgia has been repeatedly highlighted in research associated with this topic [14,20], although the most relevant premise may be the concept of “pseudo-loss” introduced by Rosenthal and Dawson’s model [25]. In this regard, the results obtained in this study corroborate the value of dysfunctional responses proposed in the first three stages of the model, although, as we have already mentioned, these elements do not seem to follow a strict chronology but appear in different combinations throughout the adaptation process, which in the end deals with accepting an unwanted situation.

The decision-making process and the resulting conflict has been especially well described on various occasions [19] and, in this regard, a relationship could be established between the patterns described for accepting institutionalization (moving towards an inescapable decision, struggling with the decision, looking for peace and quiet, and commitment to getting involved in ongoing care), and the characteristics of the decision-making conflict, developed in the field of health psychology [38] characterized by the uncertainty about the option chosen, concern about the results, hesitation, delays in making the decision, and conflict of values, among others.

Therefore, the results obtained by this work are in line with the contributions made in prior studies on the decision-making process [19], psychological implications [17,20], and factors related to the family’s adjustment to the older adult’s institutionalization [10,28].

Low scores in the subscales associated with poor adjustment prevailed in the study sample, which may be related to the fact that this is a rather homogeneous sample in terms of socio-demographic characteristics of the seniors’ relatives who have been institutionalized for a long time. On the other hand, our results confirm that family adjustment is linked to other variables of interest, such as relatives’ satisfaction with the nursing home, well-being [39], and perceived health [26], which proved to be consistent with the literature, thus supporting the validity of CAFIAR.

Specifically, the general adjustment index and subscales indicating negative adjustment reached significant co-relations in the expected direction considering depression and a negative health self-assessment; whereas the relief subscale, which points towards a positive adjustment, scored significant co-relations to well-being and a positive health self-assessment. Satisfaction regarding the nursing home negatively correlated with the score indicators of bad adjustment, whereas thankfulness towards staff significantly and positively correlated with relief. These data stand out as they highlight a fact of extraordinary importance as it is the relevance that adjustment to this institutionalization process may have on a family member’s health. In this regard, the obtained results are limited due to both deficiencies in sample size and the specificity and consistency of the employed procedure to assess such variables. We feel that it would be convenient in future investigations to address the study of the mid- and long-term consequences on the health and adjustment of the family regarding the elder’s internalization.

As for the study’s limitations, the sample is relatively small for a psychometric study, although the sample size and structure in EFA have been the subject of research for decades. Different criteria have been used to define the optimal number of cases, such as absolute amount, or the number of cases per observed variable [40]. Authors such as Catena et al. [40] suggested adding together the amount of variables or indicators observed and the number of latent constructs.

With regard to this connection, difficulties faced to obtain cooperation from the nursing home directors and relatives of the older adults admitted is relevant, which have also been reported by other authors [41]. The above may be due to the fact that, in our culture, it is commonly believed that individuals send their older relatives to live in a nursing home because they do not want to take on their care anymore [42,43], which leads to their refusal to provide information, for fear of stigma.

Limitations in the sample size and the procedure used to recruit participants (accessibility) indicate that further studies with broader and more representative samples will be necessary for the purposes of establishing scales.

Another limitation has been that, to develop the CAFIAR and explore its validation, this research study has only implemented the classic test theory. Future studies should confirm its structure validity through confirmatory analyses. There is also a need to assess its reliability by means of test–retest, also requiring longitudinal studies that contribute to understanding the adaptation process. For these reasons, we recommend researchers and clinicians who may be interested in using this questionnaire to temporarily use the percentile values of the t-scores, taking necessary precautions with respect to its interpretation.

Notwithstanding the abovementioned limitations, CAFIAR seems to be a valid tool with proper psychometric properties, which can serve as support to design and evaluate interventions, taking into account that calls for considering relatives as people in need of assistance are increasingly common. In fact, in the healthcare fields, they have been referred to as hidden patients, while many nursing homes develop intervention programs to facilitate family adjustment to the change in their role as caregivers [44,45,46]. In that regard, this paper aims to provide a measure of family adaptation to admitting older adults into a long-term nursing home that facilitates research and allows for its use in designing and evaluating intervention strategies for this population.

Taking into account the results obtained from the present research, as well as its own limitations above mentioned, we consider that future investigation lines based on this subject should be oriented towards confirming the typical structure of a family adjustment construct regarding the admission of the elder through investigation with bigger samples, applying confirmatory test techniques and in different cultural environments in order to assess the consistency of the data obtained and presented in this paper. A further aspect which ought to highlighted, and which appeals to future investigations, is the relationship between family adjustment and physical and emotional health. We consider that, in order to perform this task, it would be convenient to apply more specific instruments, as well as the possibility of carrying a temporal follow-up on the subjects of study. On this purpose, we suggest, in face of future investigations, to apply measures of emotional closeness between the elder and their family, as an aspect which has not been fully explored in the present paper.

## 5. Conclusions

In conclusion, CAFIAR is an assessment instrument suitable to evaluate family adaptation to admitting an older adult to a nursing home. This is comprised of 15 items, with a Cronbach’s alpha coefficient of 0.74, including 3 factors: Factor 1 (Unease due to admitting the older adult to the nursing home), Factor 2 (Relief), and Factor 3 (Nostalgia and concern for the older adult). Thus, we suggest obtaining a general adjustment index as well.

This is a suitable instrument for research and work applied to the relatives of older adults institutionalized in long-term nursing homes.

## Figures and Tables

**Table 1:** Socio-demographic characteristics of the family members participating in the study.

Socio-Demographic Characteristics	*n*	%
Sex		
Men	46	34.3
Women	88	65.7
Total	134	100
Relationship		
Spouse	8	6
Son/daughter	95	70.9
Daughter/son-in-law	8	6
Grandchild	5	3.7
Nephew/niece	7	5.2
Other	11	8.2
Total	134	100
Care task		
Primary caregiver	58	43.3
Eventual caregiver	28	20.9
Shared care tasks	34	25.4
No relevant care tasks	14	10.4
Total	134	100
Marital status		
Married	108	80.6
Divorced/separated	7	5.2
Widowed	7	5.2
Single	12	9
Total	134	100
Employment status		
Domestic work	24	17.9
Part-time work outside home	14	10.4
Full-time work outside home	45	33.6
Unemployed	11	8.2
Student	2	1.5
Retired	29	21.6
Other	9	6.7
Total	134	100
Level of education		
No formal education	7	5.2
Primary school	53	39.6
Middle school	46	34.3
High school	25	18.7
Lost	3	2.2
Total	134	100

**Table 2:** Test and item specifications.

Construct to be assessed: Family adjustment to an older adult’s admission to a nursing home Definition: This is a multi-dimensional construct referring to the feelings experienced by the interviewees at a given moment, throughout their personal process of adapting to an older family member’s admission to a nursing home.
The relative’s adjustment to the senior’s admission to a nursing home is understood as a complex process, which may lead to conflicting experiences and feelings of relief, guilt, sadness, nostalgia, and concern for the older adult’s well-being.
This process can be more or less intense and last for a longer or shorter time depending on the closeness of the relationship, and especially the older adult’s main caregiver, if any of the senior’s family members served that role before this situation.
Instrument: Family adjustment in admitting an older adult to a nursing home questionnaire.
Objective: Assessing the relative’s adaptation.
Application: Research, design, and evaluation of the impact that individual and collective interventions have on institutionalized older adult’s family caregivers.
Expected characteristics of the instrument:
A questionnaire that may be self-administered or applied through interviews. Answers should be provided using a Likert scale regarding intensity (none to a lot), as it seeks to assess the relative’s level of adjustment at a given time, in general and in terms of the various aspects of said adaptation. These values must be useful for their application to repeated measures research designs. The instrument should be concise such that it can be repeatedly used quickly with minimal time and effort. The instrument should be simple to allow its self-administration in individuals with poor cultural training, where the circumstances of the study demanding its application so require.
Questionnaire format: It can be applied either individually or collectively, on paper or via computer, during the family visit to the institution or at home, remotely (online) or via phone call.
Recipients: Educated adults who are capable of understanding the questions.
Task: Those interviewed must state to what degree a set of statements reflect their current feelings concerning their relative’s admission to the nursing home.
Item characteristics: Expressions regarding positive and negative feelings. A 5-point Likert scale (No, A little bit, Moderately, A lot, and Completely).
Content areas to assess
Component 1: Unease due to an older adult’s admission to a nursing home.
Objective: To evaluate the reactions or signs of dysphoria (doubts regarding the suitability of the decision made, feelings of guilt, acceptance of the situation, internal disagreement) experienced by the family member as a result of the whole process, which entails carrying out the decision to move an aging relative into a nursing home.
Component 2: Relief of burden
Objective: To evaluate feelings of relief, personal life rearrangement, and enjoyment of activities that the individual was not able to participate in before the older adult entered the nursing home.
Component 3: Nostalgia/feelings of loss (pseudo-loss) and concern for the older adult
Objective: To evaluate feelings of nostalgia, longing and concern for the institutionalized older adult.

**Table 3:** Items included in the questionnaire’s 22-item version.

Questionnaire’s Item
1. I feel guilty because my relative has been admitted the nursing home.
2. I feel I should have not put my relative in the nursing home.
3. I miss my relative.
4. I feel I can make more time for myself.
5. I feel relieved.
* 6. I enjoy visiting my relative.
7. I am worried that the nursing home staff will not take good care of my family.
8. I have enjoyed doing new activities.
* 9. I feel upset with my relative because of his/her lack of support in avoiding institutionalization.
10. I perform better in my day-to-day tasks.
* 11. I visit the nursing home less often, but I enjoy the visits more than before.
12. I wish my relative could come back home.
13. I believe my relative’s health will worsen in the short term.
* 14. I feel bad every time I visit my relative.
15. I feel like nobody else is capable of taking care of my relative.
* 16. I feel satisfied with the frequency of my visits to my relative.
* 17. My relative makes me feel guilty every time I visit him/her.
18. I have enjoyed returning to the activities I was not able to do before.
* 19. I feel upset because my relative is not doing enough to adapt to living in the nursing home.
20. I think that my relative’s health will worsen due to his/her stay in the nursing home.
21. I have adapted to the change this situation entailed for my family relationships.
22. I have accepted the fact that my relative is living in the nursing home now.

* Items removed in final version.

**Table 4:** Configuration matrix.

Items	Factor		
	1	2	3
22. I have accepted the fact that my relative is living in the nursing home.	−0.99		
21. I have adapted to the change this situation entailed in my family relationships.	−0.68		
2. I feel I should have not put my relative in the nursing home.	0.58		
1. I feel guilty because my relative has been admitted to the nursing home.	0.58		
15. I feel like nobody else is capable of taking care of my relative.	0.37		,
10. I perform better in my day-to-day tasks.		0.76	
18. I have enjoyed returning to the activities I was not able to do before.		0.73	
23. I feel like I can make more time for myself.		0.71	
8. I have enjoyed doing new activities.		0.64	
24. I feel relieved.		0.50	
3. I miss my relative.			0.60
7. I am worried that the nursing home staff will not take good care of my family.			0.58
13. I believe my relative’s health will worsen in the short term.			0.53
12. I wish my relative could come back home.			0.47
20. I think that my relative’s health will worsen due to staying in a nursing home.			0.43

Extraction method: Principal axis factoring. Rotation method: Oblimin with Kaiser normalization.

**Table 5:** Correlations between the scores of the CAFIAR subscales (15-item version) and CES-D, well-being indicators, health self-assessment, and satisfaction with the nursing home.

	Factor 1 Dissatisfaction with the Decision and Guilt	Factor 2 Relief and Life Rearrangement	Factor 3 Nostalgia and Concern for the Older Adult	General Adjustment Index (GAI)
CES-D	0.32 **	0.17	0.40 **	0.25 **
Relief	−0.57 **	0.24 **	−0.46 **	−0.56 **
Satisfaction	−0.49 **	0.21 *	−0.48 **	−0.52 **
Optimism	−0.32 **	0.51 **	−0.11	−0.42 **
Energy	−0.25 **	0.51 **	−0.15	−0.40 **
Happiness	−0.46 **	0.47 **	−0.18 *	−0.46 **
I feel like my health condition has worsened.	0.35 **	−0.18 *	0.22 *	0.34
Physically, I feel fine.	−0.31 **	0.27 **	−0.21 *	−0.35 **
I believe my health will improve.	0.02	0.48 **	0.00	−0.20 *
I am grateful for the aid provided by the nursing home staff.	−0.49 **	0.23 **	−0.52 **	−0.54 **
I believe the staff is doing a good job.	−0.49 **	0.11	−0.53 **	−0.44 **
I think residents are satisfied with the nursing home staff.	−0.44 **	0.07	−0.48 **	−0.50 **
I think the nursing home staff cares about patients.	−0.48 **	0.12	−0.52 **	−0.50 **
I trust the nursing home staff.	−0.48 **	0.12	−0.53 **	−0.60 **

* *p* = 0.01; ** *p* = 0.00.

**Table 6:** CAFIAR’s statistical data, 15-item version.

	Raw Score Factor 1		T-Score Factor 1	Raw Score Factor 2	T-Score Factor 2	Raw Score Factor 3	T-Score Factor 3	General Adjustment Index (GAI)	General Adjustment T-Score
**Average**	8.99		49.58	14.59	49.54	11.56	48.37	5.90	49.30
Median	7.00		44.58	15.00	50.45	11.00	47.05	4.00	47.29
Mode	5.00		39.41	15.00	50.45	5.00	33.04	0.00	43.04
Standard dev.	4.62		11.93	4,83	10.60	5.01	11.69	10.96	11.64
Asymmetry	1.59			0.03		0.63		0.95	
Asymmetry stand. error	0.21			0.21		0.21		0.22	
Kurtosis	1.82			−0.74		−0.59		0.56	
Kurtosis stand. error	0.42			0.42		0.42		0.43	
Minimum	5.00		39.41	5.00	28.49	5.00	33.04	−14.00	28.16
Maximum	25.00		91.03	25.00	72.40	23.00	75.06	38.00	83.42
Percentile	5	5.00	39.40	5.00	32.88	5.00	33.04	33.04	33.79
	10	5.00	39.40	5.20	35.08	5.20	33.51	33.51	36.66
	30	6.00	41.90	8.00	42.98	8.00	40.05	40.05	43.04
	50	7.00	44.50	11.00	50.45	11.00	47.09	47.05	47.29
	70	9.00	49.70	13.00	54.84	13.00	51.72	51.72	52.82
	90	17.00	70.30	20.00	63.62	20.00	68.06	68.06	68.97
	95	20.00	78.10	21.40	65.81	21.40	71.32	71.32	73.54

## Appendix A

**Table A1:** CAFIAR’s final version.

			No	A Little Bit	Moderately	A Lot	Completely
F1	1.	I feel guilty because my relative has been admitted the nursing home					
F1	2.	I feel like I should not have put my relative in the nursing home					
F3	3.	I miss my relative					
F2	4.	I feel like I can make more time for myself					
F2	5.	I feel relieved					
F3	6.	I am worried that the nursing home staff will not take good care of my family					
F2	7.	I have enjoyed doing new activities					
F2	8.	I perform better in my day-to-day tasks					
F3	9.	I wish my relative could come back home					
F3	10.	I believe my relative’s health will worsen in the short term					
F1	11.	I feel like nobody else is capable of taking care of my relative					
F2	12.	I have enjoyed returning to activities I was not able to do before					
F3	13.	I think that my relative’s health will worsen due to staying in the nursing home					
F1	14.	I have adapted to the change this situation entailed in my family relationships					
F1	15.	I have accepted the fact that my relative is living in the nursing home now					
